# Supplementary material for: Intrinsic cell rheology drives junction maturation
Source: Nat Commun. 2022 Aug 17;13:4832. doi: 10.1038/s41467-022-32102-9 (PMC9385638; doi:10.1038/s41467-022-32102-9)
Supplement: Supplementary file 1 — Supplementary Information [file 41467_2022_32102_MOESM1_ESM.pdf]

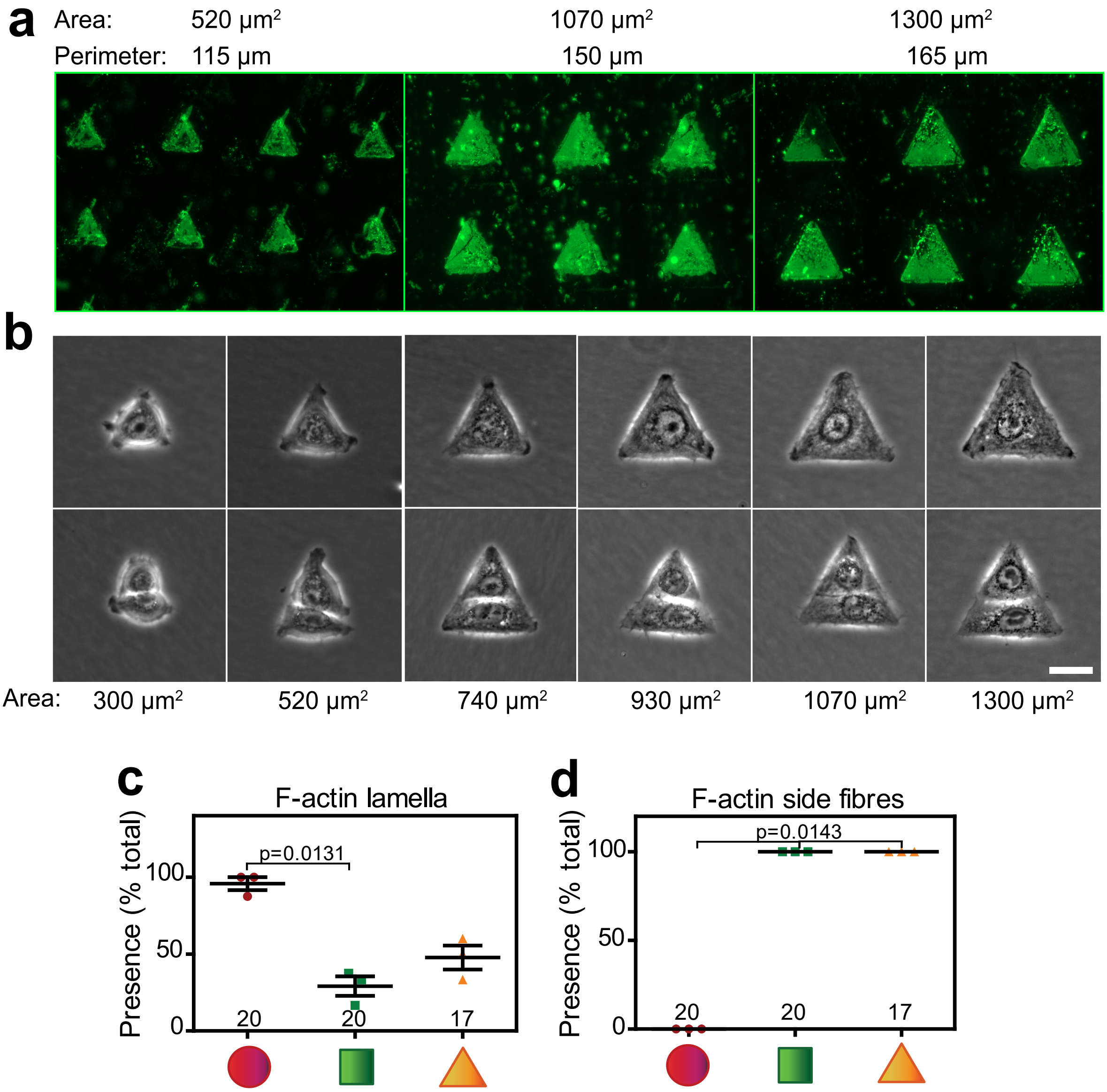


**Supplementary Fig. 1: Optimization of keratinocytes spreading on micropatterns and geometric shape-dependent cytoskeletal organization**

Different geometric shapes as circles, squares and triangles of different sizes were used to test keratinocyte attachment and spreading. **a**, Efficiency of fibronectin coating of triangular shapes of different sizes. **b**, Keratinocytes attached as single cell or cell pairs on the different sizes of triangular shapes. Phase contrast images were taken of normal keratinocytes after 24 hours growth. For further experimentation, images of micropatterns of 1300 $\mu m^2$ containing two cells were selected for analysis. **c-d**, Quantification of the percentage of micropatterns in which cells display lamella (**c**) or F-actin bundles as side fibres (**d**). In **c-d**, micropattern geometries are represented as red circles, green squares and orange triangles. Number of cell pairs (**c-d**) quantified is shown on top of each geometric shape (N=3). Mean values and error bars (SEM) are shown. Statistical analysis was done by one-way ANOVA, followed by Kruskal-Wallis post-hoc with Benjamin, Krieger and Yekutieli multiple comparisons tests. Scale bars = 20 $\mu$m. Source data are provided as a Source Data file.

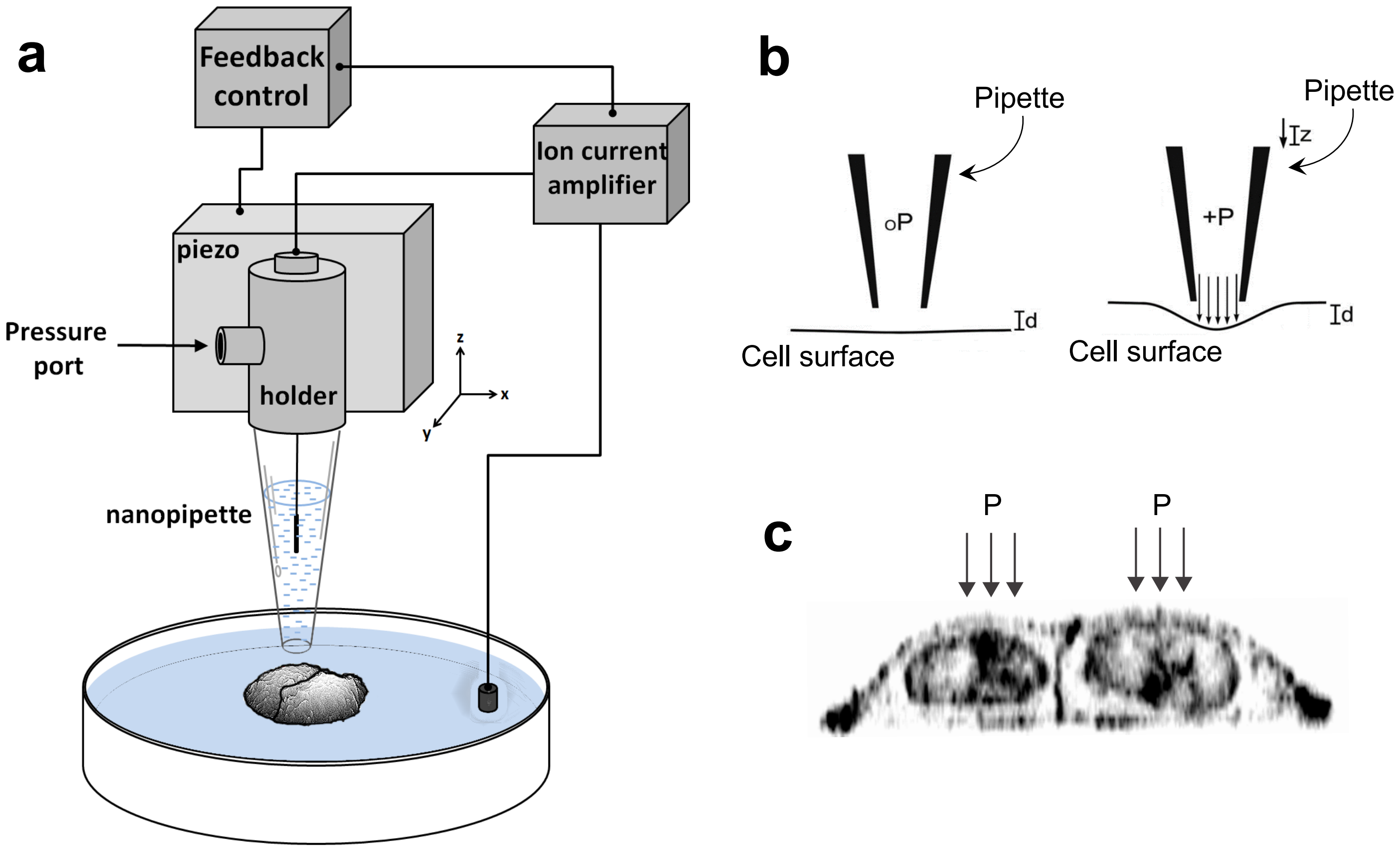


**Supplementary Fig. 2: Measurement of biophysical parameters with SICM.** Methodology of the SICM technique. **a**, Schematic of the SICM system set-up containing a pressure port through which a transient pressure is applied. **b**, Non-contact alignment of the pipette with the cell surface to obtain topographical maps (left) and displacement of the surface upon pressure application (right). **c**, Sites of measurement of pressure on the cell surface (i.e., the apices of individual cells).

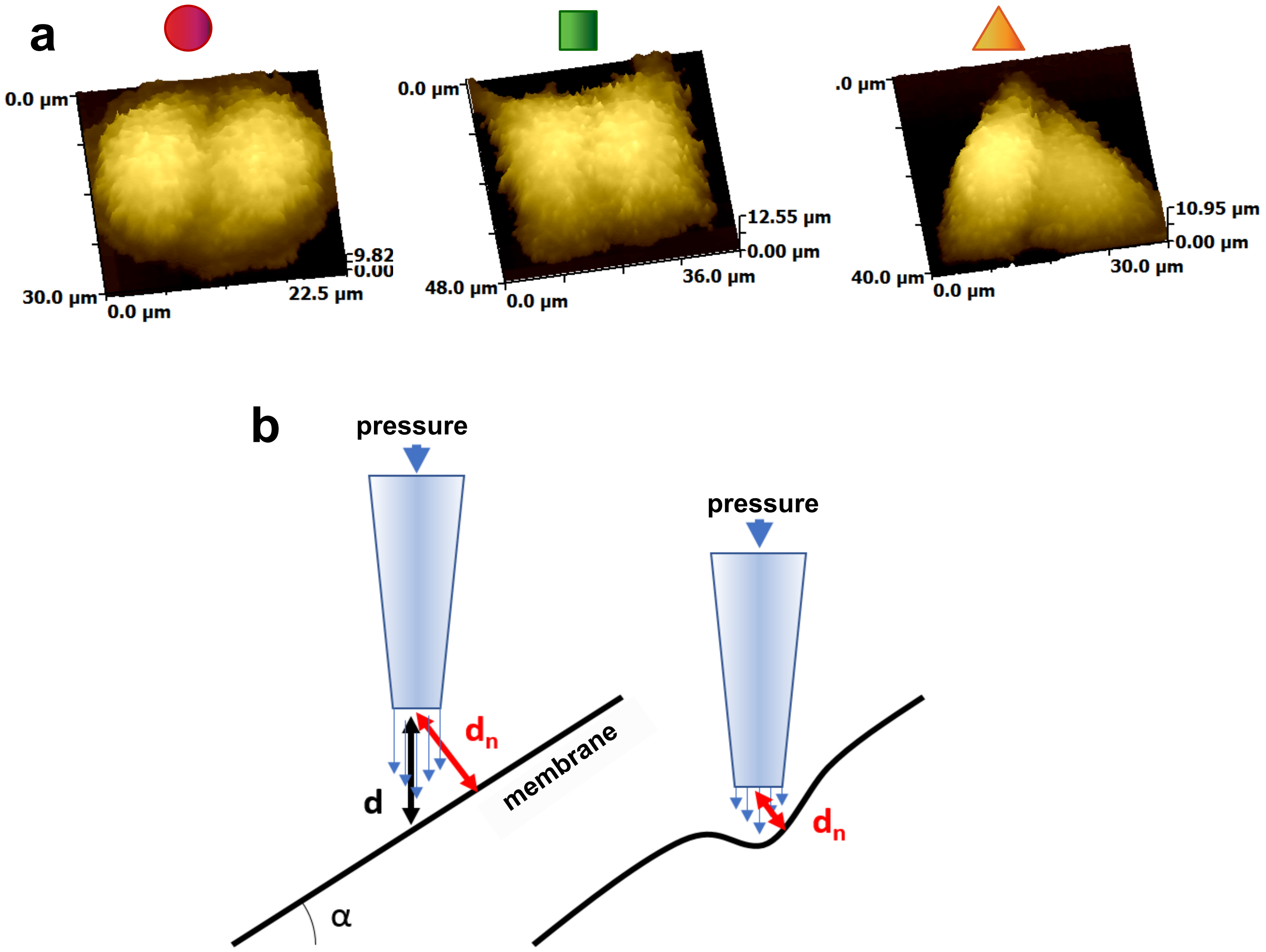


**Supplementary Fig. 3: Principle of slope correction in the measurement of Young's modulus.**

**a**, Using a slope correction method, topographical profiles obtained with SICM for cell doublets on circle, square and triangles were used to measure cell height, area, and volume. Micropattern geometries are represented above images as red circles, green squares and orange triangles. **b**, The displacement of membrane due to pressure application is likely to be overestimated if the probe is not perpendicular to the membrane. This may lead to underestimation (lower values) of Young's modulus, particularly at the edges of cells. It has been reported that the effect of slope can be minimised if the nanopipette-surface distance is measured in a direction perpendicular to the surface ($d_n$) instead of the z-axis direction ($d$):[1]

$$d \quad = d \quad \cos\alpha$$

To correct for the effect of slope, local slope (represented by the angle α in the equation above) was first estimated from the topography image. The displacement values measured during the application of the pressure were then multiplied by local value of $\cos\alpha$ Displacement values corrected for local slope were then used to calculate Young's modulus based on the method described previously.[2]

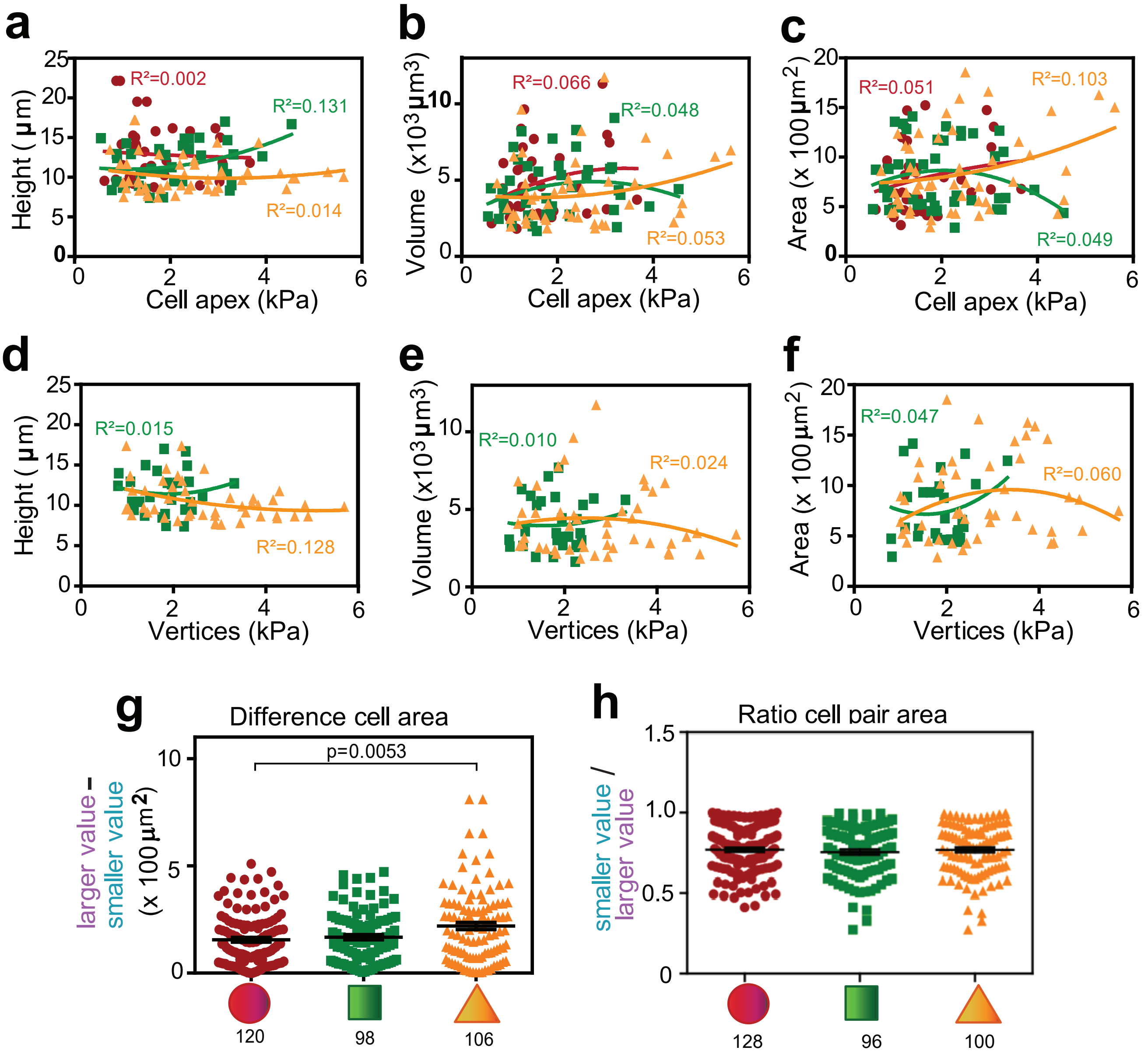


**Supplementary Fig. 4: Correlations of cortex stiffness (cell apex and vertices) with the height, volume and area of each cell.**

**a-f**, Each neighbour from a cell pair sharing a micropattern of different shapes (circular, squares of triangles) were scanned with SICM and its biophysical properties measured and compared to its companion. Values obtained for each cell were then plotted to assess potential correlations. Stiffness measured at the cell apex (**a-c**) or at the vertices (**d-f**) was plotted against height (**a**, **d**), volume (**b**, **e**) or area (**c**, **f**). **g-h**, Cell area of each neighbour sharing a micropattern was measured. Graphs show the difference between the largest and smallest area (**g**) or the ratio between the smallest and largest area (**h**). Different micropattern geometries are represented as a line inside graphs (**a-f**) below graphs (**g-h**) as red (circles), green (squares) and orange (triangles) lines or shapes. Each point represents one micropattern of different geometry. Mean values and error bars (SEM) are shown (**g-h**). Goodness of fit ($R^2$) is shown for each curve (**a-f**) or statistical analyses performed using one-way ANOVA, followed by Kruskal-Wallis post-hoc test and with Benjamin, Krieger and Yekutieli test to control for False Discovery Rate. N=7, **a-f**; N=4, **g-h**. Source data are provided as a Source Data file.

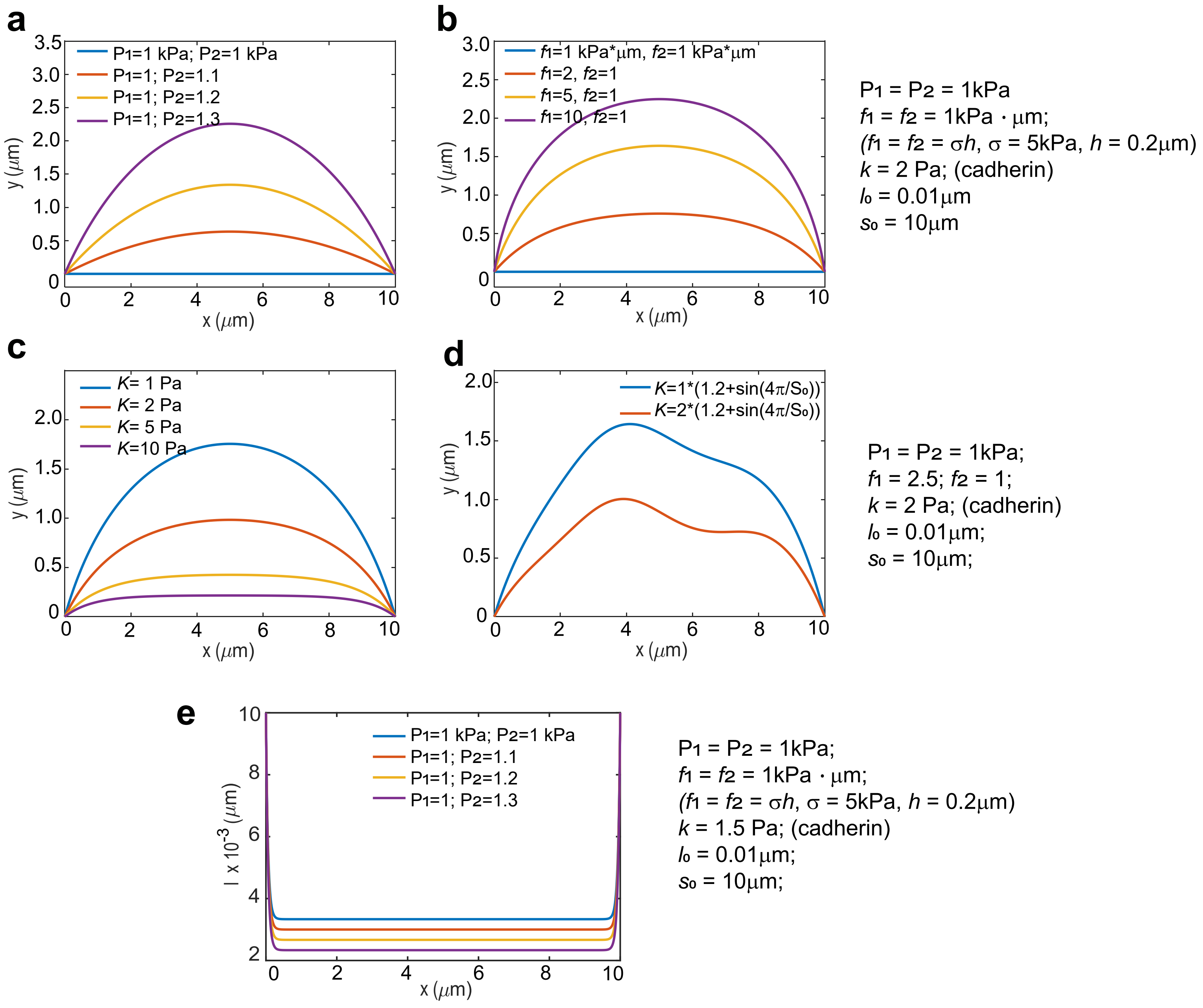
a
P1=1 kPa; P2=1 kPa
P1=1; P2=1.1
P1=1; P2=1.2
P1=1; P2=1.3
y (μm)
x (μm)
b
f1=1 kPa*μm, f2=1 kPa*μm
f1=2, f2=1
f1=5, f2=1
f1=10, f2=1
y (μm)
x (μm)
P1 = P2 = 1kPa
f1 = f2 = 1kPa · μm;
(f1 = f2 = σh, σ = 5kPa, h = 0.2μm)
k = 2 Pa; (cadherin)
l0 = 0.01μm
s0 = 10μm
c
K= 1 Pa
K= 2 Pa
K= 5 Pa
K=10 Pa
y (μm)
x (μm)
d
K=1*(1.2+sin(4π/S0))
K=2*(1.2+sin(4π/S0))
y (μm)
x (μm)
P1 = P2 = 1kPa;
f1 = 2.5; f2 = 1;
k = 2 Pa; (cadherin)
l0 = 0.01μm;
s0 = 10μm;
e
P1=1 kPa; P2=1 kPa
P1=1; P2=1.1
P1=1; P2=1.2
P1=1; P2=1.3
l x 10-3 (μm)
x (μm)
P1 = P2 = 1kPa;
f1 = f2 = 1kPa · μm;
(f1 = f2 = σh, σ = 5kPa, h = 0.2μm)
k = 1.5 Pa; (cadherin)
l0 = 0.01μm;
s0 = 10μm;

**Supplementary Fig. 5: Model fitting reproduces the various scenarios of junction shape predicted in our model** (Figure 3). Graphs show computed cell-cell junction shapes based on our model equations using parameter values estimated based on physical values. Data show that the effective stiffness density of cadherin bonds (*k*) has a strong effect on the boundary shape. The effects of the different parameters on the shape of computed junctions are: **a**, varying the pressure in one of the cells ($P_2$); **b**, varying ($f_1$ ), which combined cell surface tension in one of the cells ($f_2$ ) which includes cortical thickness and the cortical stress; **c**, varying the effective stiffness density of the E-cadherin bonds (*k*); **d**, spatial variation of (*k*) along junctions, generating an undulated shape and **e**, assessing the intergap *l*(s) contribution to the force balance at contacting interface. Individual parameter values and shape output are shown inside each graph. Parameters values written on the right refer to graphs on top row (**a**-**b**), middle row (**c**-**d**) or bottom row (**e**), respectively. Different coloured curves in each graph represent the changes in interface caused by variations of the respective parameters as outlined inside graphs. Source data are provided as a Source Data file.

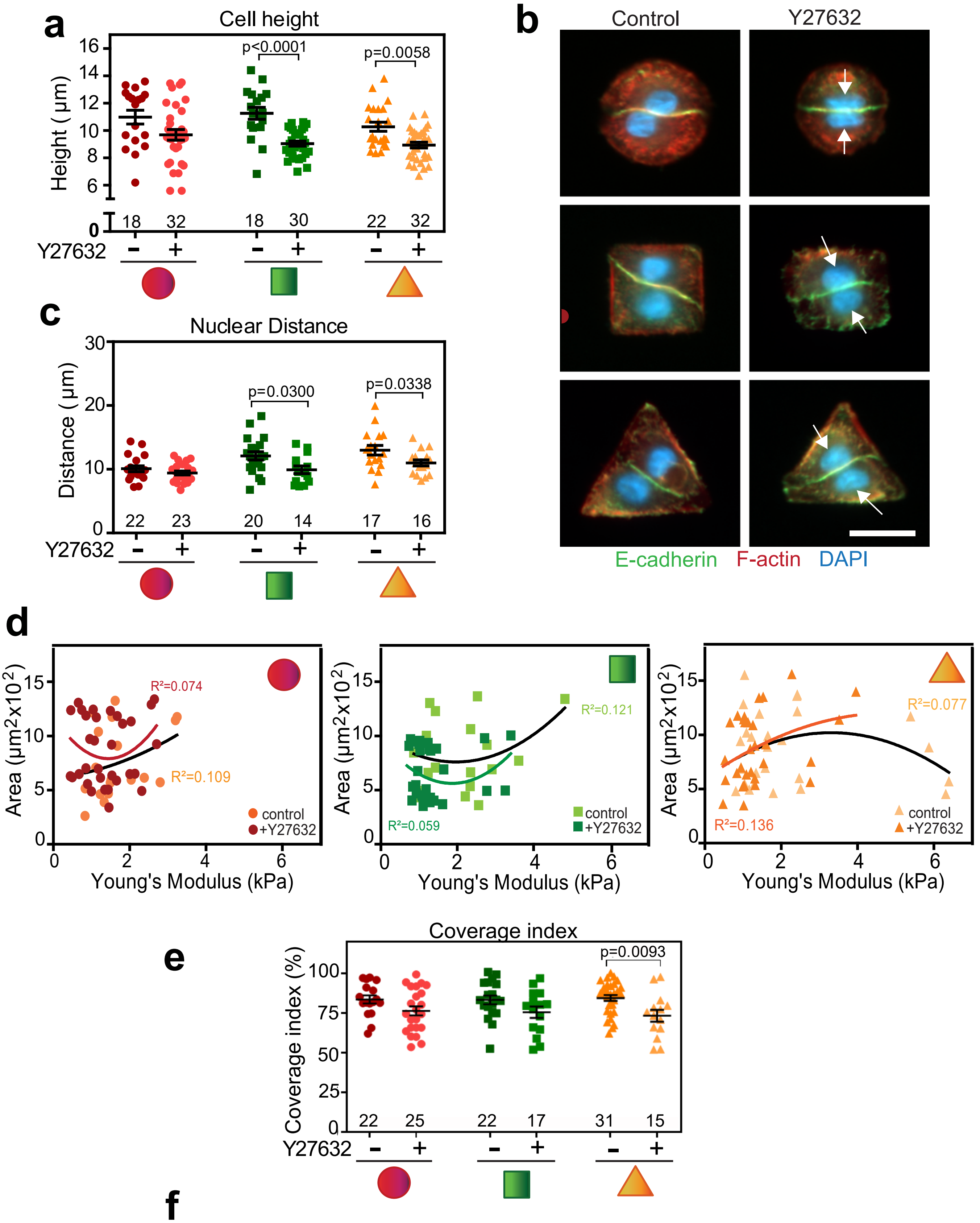


**f**

| | α1 | α2 | α3 | α4 | α5 | α6 |
|---|---|---|---|---|---|---|
| control | 1 | 1.862 | 0.34495 | 1.48902 | 7069.71 | 0.001 |
| Y27632 | 1 | 1.862 | 0.64948 | 0.46151 | 7069.71 | 0.001 |

**Supplementary Fig. 6: Inhibition of cell contractility alters biophysical properties of cell doublets on geometric shapes.**

Cell doublets confined on different geometries were treated with or without Y27632 and processed using SICM (**a**, **d**) or immunofluorescence staining for E-cadherin, F-actin or nuclei (**b**-**c**). **a**, Topographical maps obtained with SICM (Fig.S4a) were used to calculate height at the cell apex before and after treatment with Y27632. **b**, Representative images of control and treated cells show that relaxation leads to more curvilinear junctions and the collapse of the nucleus towards cell-cell contacts (arrows). **c**, Distance between nuclei of neighbouring cells sharing a micropattern was measured using FIJI. **d**, Cortical stiffness (Young's Modulus at cell apex) does not correlate with cell area. **e**, Y27632 treatment reduces the percentage of available contacting interface length that contains E-cadherin staining (coverage index). **f**, Parameters calculated during modelling of the influence of cortical relaxation on junction linearity (see text for more details). The parameters are assumed to be spatially constant. Different micropattern geometries are represented inside (**d**) or below (**a, c, e**) graphs as red (circles), green (squares) and orange (triangles) lines or shapes Scale bar = 20μm. Number of samples quantified is shown on top of each geometric shape (N=3, **a**-**c**, **e**; N=7, **d**). Mean values and error bars (SEM) are shown. Statistical analysis was done by Mann-Whitney test (unpaired, two-tailed) (**a, c, e**). Source data are provided as a Source Data file.

## Supplementary note

### A Mechanical Model of Cell-Cell Junction:

For typical mammalian cells, the internal hydraulic pressure is slightly higher than the extracellular environment, due to excess osmotic pressure in the cytoplasm.[1] When the cell shape is steady, i.e., not changing in time, forces at the cell surface must be balanced. The following force balance conditions can be derived for the cell membrane in the normal and tangent directions

$$T(\nabla\cdot\boldsymbol{n}) = \boldsymbol{n}\cdot\boldsymbol{\sigma}|_S\cdot\boldsymbol{n} + P \quad \text{(Normal component)}$$
$$(\nabla T)\cdot\boldsymbol{t} = -\boldsymbol{t}\cdot\boldsymbol{\sigma}|_S\cdot\boldsymbol{n} \quad \text{(Tangent component)} \quad (1)$$

where $T$ is the tension in the cell membrane and $(\boldsymbol{n},\boldsymbol{t})$ are unit normal and tangent vectors at the membrane surface $S$. $\boldsymbol{\sigma}$ is the stress tensor in cell cortex and $P$ is hydraulic pressure in the cytoplasm. $(\nabla\cdot\boldsymbol{n}) = 2H$ where $H$ is the mean curvature of $S$. It was shown by solving for an active fluid model of the cell cortex, $\boldsymbol{n}\cdot\boldsymbol{\sigma}|_S\cdot\boldsymbol{n}$ is dominated by the active stress (myosin contraction) in the cortex, passive stress from the flow of the actin network can be neglected (see SI material of [2]). Therefore,

$$T(\nabla\cdot\boldsymbol{n}) = -\sigma_a h(\nabla\cdot\boldsymbol{n}) + P$$

where $\sigma_a$ is the tangential component of the active stress from myosin contraction. Moreover, $\sigma_a h \gg T$ and the membrane tension is generally small when compared to active stress. If the cortical stress is mostly from myosin contraction, the result is a Young-Laplace like relation for the cell surface

$$P - 2(\sigma_a h)H = 0$$

When two cells are in contact, cells can form E-cadherin bonds that spans the cell surface (Fig. 4A in the main text). We assume these bonds are perpendicular to the cell membrane and contributes to the normal force balance condition. Therefore

$$P - 2(\sigma_a h)H + k\varepsilon = 0$$

where $\epsilon = (l - l_0)/l_0$ is the strain in the E-cad bonds, or the separation distance between 2 cell membranes. Note that here, we do not consider possible mechanical interaction between cortical actin and E-cad bonds. This could potentially change $\epsilon$ and $\sigma_a$. Here, we treat these quantities as unknown independent parameters. Thus, writing this for membranes of cells 1 and 2, we have Eq. (1) in the main text.

$$\begin{aligned} P_1 - f_1 H_1 + k\varepsilon = 0 \\ P_2 + f_2 H_2 + k\varepsilon = 0 \end{aligned} \qquad (2)$$

where we have redefined $2\sigma_a H$ as $f_i$ for $i = (1,2)$.

In 2D, the cell surface is an 1D line and can be written as

$$\boldsymbol{r}_1(s) = \boldsymbol{r}(s) + \frac{l(s)}{2}\boldsymbol{n}$$
$$\boldsymbol{r}_2(s) = \boldsymbol{r}(s) - \frac{l(s)}{2}\boldsymbol{n}$$

where $s$ is an arclength along the cell-cell junction line $\boldsymbol{r}$. The cell membrane curvatures are approximately $H_1 = H + l''/2$ and $H_2 = H - l''/2$, where $H$ is the mean curvature of the central line $\boldsymbol{r}(s)$. Adding the two equations in Eq. 2 after dividing by $f_i$ cancels $H$, and gives the governing equation for E-cadherin bond strain:

$$\frac{\partial^2 l}{\partial s^2} - \left(\frac{1}{f_1} + \frac{1}{f_2}\right) k \frac{l - l_0}{l_0} - \left(\frac{P_1}{f_1} + \frac{P_2}{f_1}\right) = 0 \qquad (3)$$

This is a fully closed ODE for $l(s)$ that can be solved with 2 boundary conditions. We assume zero strain at the ends of the cell-cell contact, i.e., $l(0) = l(L) = l_0$. Once l is solved, we can use Eq. 2 again to obtain

$$H_1 = \frac{1}{f_1}\left(P_1 + k\frac{l - l_0}{l}\right)$$
$$H_2 = \frac{1}{f_2}\left(P_2 + k\frac{l - l_0}{l}\right)$$
$$H = \frac{1}{2}(H_1 + H_2)$$

For cells on the small micropatterns, due to confinement, the cell nucleus may directly impinge on the cell-cell junction. The force from the nucleus compression may be more important than the hydraulic pressure of the cytoplasm. Therefore, $P_{1,2}$ may be coming from nucleus and is proportional to the distance from the nucleus to the cell-cell junction. Therefore, we model $P_{1,2}$ as $a_{1,2}/ND$ where $a_{1,2}$ are unknown parameters and ND is the distance from the junction to

the nucleus. $f_{1,2}$ are proportional to contractile stress from myosin activity in the cortex. We assume this is proportional to the F-actin intensity detected at the cell junctions, i.e., $f_{1,2} = a_{3,4} I_{actin}$ where $a_{3,4}$ are unknown parameters. The E-cad bond stiffness density, $k$, is proportional to the E-cad intensity detected at the cell junction, i.e., $k = a_5 I_{Ecad}$, where $a_5$ is an unknown parameter. Finally, $l_0 = a_6$ is another parameter.

For given parameters $a_1$ to $a_6$, which are constants and independent of $s$, and measured F-actin and E-cad intensities and nuclear position, which are functions of $s$, we can compute the cell-cell junction shape as given above. Some results are given in Fig. 4 in the main text. The parameters $a_1$ to $a_6$ are not dimensionless, e.g., $a_1$ relates a distance to a pressure. In our work, we simply treat them as fitting parameters. These parameters will depend on the experimental condition, and the type of cell in question. Therefore, the parameters $a_1$ to $a_6$ do not have physical meaning. However, quantities such as $P_{1,2}$ and $f_{1,2}$ do have physical meaning, but they are not directly measured. The parameters $a_1$ to $a_6$ simply connects experimental measurements (image intensities) to physical quantities.

## Supplementary references


1. Thatenhorst, D., Rheinlaender, J., Schaffer, T.E., Dietzel, I.D. & Happel, P. Effect of sample slope on image formation in scanning ion conductance microscopy. *Anal. Chem.* **86**, 9838-9845 (2014).
2. Rheinlaender, J. & Schäffer, T.E. Mapping the mechanical stiffness of live cells with the scanning ion conductance microscope. *Soft Matter* **9**, 3230 (2013).
3. Li, Y., Konstantopoulos, K., Zhao, R., Mori, Y. & Sun, S.X. The importance of water and hydraulic pressure in cell dynamics. *J. Cell Sci.* **133** (2020).
4. Tao, J. & Sun, S.X. Active Biochemical Regulation of Cell Volume and a Simple Model of Cell Tension Response. *Biophys. J.* **109**, 1541-1550 (2015).
