# Supplementary material for: Intrinsic cell rheology drives junction maturation
Source: Nat Commun. 2022 Aug 17;13:4832. doi: 10.1038/s41467-022-32102-9 (PMC9385638; doi:10.1038/s41467-022-32102-9)
Supplement: Supplementary file 3 — Description of Additional Supplementary Files [file 41467_2022_32102_MOESM3_ESM.pdf]

Description of Additional Supplementary Files

File Name: Supplementary Data 1
Description: Code to model biophysical variables affecting contacting interfaces.
