# Supplementary material for: Intrinsic cell rheology drives junction maturation
Source: Nat Commun. 2022 Aug 17;13:4832. doi: 10.1038/s41467-022-32102-9 (PMC9385638; doi:10.1038/s41467-022-32102-9)
Supplement: Supplementary file 5 — Reporting Summary [file 41467_2022_32102_MOESM5_ESM.pdf]

nature portfolio

Corresponding author(s): Vania Braga & Julia Gorelik

Last updated by author(s): 25/05/2022

Reporting Summary

Nature Portfolio wishes to improve the reproducibility of the work that we publish. This form provides structure for consistency and transparency in reporting. For further information on Nature Portfolio policies, see our Editorial Policies and the Editorial Policy Checklist.

Statistics

For all statistical analyses, confirm that the following items are present in the figure legend, table legend, main text, or Methods section.

n/a	Confirmed	
☐	☒	The exact sample size (*n*) for each experimental group/condition, given as a discrete number and unit of measurement
☐	☒	A statement on whether measurements were taken from distinct samples or whether the same sample was measured repeatedly
☐	☒	The statistical test(s) used AND whether they are one- or two-sided<br>*Only common tests should be described solely by name; describe more complex techniques in the Methods section.*
☒	☐	A description of all covariates tested
☐	☒	A description of any assumptions or corrections, such as tests of normality and adjustment for multiple comparisons
☐	☒	A full description of the statistical parameters including central tendency (e.g. means) or other basic estimates (e.g. regression coefficient) AND variation (e.g. standard deviation) or associated estimates of uncertainty (e.g. confidence intervals)
☐	☒	For null hypothesis testing, the test statistic (e.g. *F*, *t*, *r*) with confidence intervals, effect sizes, degrees of freedom and *P* value noted<br>*Give P values as exact values whenever suitable.*
☒	☐	For Bayesian analysis, information on the choice of priors and Markov chain Monte Carlo settings
☒	☐	For hierarchical and complex designs, identification of the appropriate level for tests and full reporting of outcomes
☒	☐	Estimates of effect sizes (e.g. Cohen's *d*, Pearson's *r*), indicating how they were calculated

Our web collection on statistics for biologists contains articles on many of the points above.

Software and code

Policy information about availability of computer code

Data collection	Data was collected using commercial software running the microscopes wide-field and confocal in our Microscopy facility (FILM; i.e., Zen software [Carl Zeiss]). For the SICM experiments, the topographical data were collected using SICM scanner software generating images in developed previously in Dr Pavel Novak's lab and published elsewhere (DOI: 10.1039/c8nr03870h; DOI: 10.1039/d0nr02474k; DOI: 10.1073/pnas.1917171117).
Data analysis	SICM analyses used SICM Image Viewer. Custom-made code for analysing SICM images is available from https://github.com/PavelNo/SICMImageViewer. Most of the data processing and analyses of fluorescence images were done using FIJI. Analyses of some experiments used custom-made software, Junction Mapper, as per our publication (DOI: 10.7554/eLife.45413). The software and instructions can be downloaded and accessed in https://dataman.bioinformatics.ic.ac.uk/junction_mapper/ or DOI: 10.5281/zenodo.6563424. Junction Mapper code is deposited in DOI: 10.5281/zenodo.6563424 https://github.com/ImperialCollegeLondon/Junction_Mapper.

For manuscripts utilizing custom algorithms or software that are central to the research but not yet described in published literature, software must be made available to editors and reviewers. We strongly encourage code deposition in a community repository (e.g. GitHub). See the Nature Portfolio guidelines for submitting code & software for further information.

Data

Policy information about availability of data

All manuscripts must include a data availability statement. This statement should provide the following information, where applicable:

- Accession codes, unique identifiers, or web links for publicly available datasets
- A description of any restrictions on data availability
- For clinical datasets or third party data, please ensure that the statement adheres to our policy

The datasets generated during and/or analysed during the current study added to the Source data file or deposited in Figshare (https://doi.org/10.6084/m9.figshare.c.6094764). Any other information or reagent request are available from the corresponding authors on reasonable request.

Field-specific reporting

Please select the one below that is the best fit for your research. If you are not sure, read the appropriate sections before making your selection.

☒ Life sciences ☐ Behavioural & social sciences ☐ Ecological, evolutionary & environmental sciences

For a reference copy of the document with all sections, see nature.com/documents/nr-reporting-summary-flat.pdf

Life sciences study design

All studies must disclose on these points even when the disclosure is negative.

Sample size	No sample size calculations were performed prior to the experiments; the limitation of data collection was cell attachment on micropatterns. Primary cells attached at random on the micropatterns and all suitable images were collected in an experiment (i.e., cell pairs and fully spread covering the whole surface of the geometric shape - please see below). Across the research project, at least three biological replicates were made, and if necessary, further biological replicates were done. Once the phenotype of each replicate was confirmed, samples were pooled for analyses (number of micropatterns or individual cells depicted in each graph). For standard image analyses using automated image acquisition in wide-field or confocal analyses, data was acquired per micropattern (20 < n > 102) or per cell (twice the number of micropatterns). In case of more laborious and challenging analyses, a minimum of samples were analysed across different replicates for FRAP (12 > n > 24) and SICM experiments in normal cells (18 > n > 48) or with pharmacological inhibition (9 > n > 32).
Data exclusions	Cells attached at random as one, two or more cells. Only micropatterns that contained cell doublets fully spread on the fibronectin coated geometric shape were selected for quantification. Pre-established criteria for exclusion were: images out of focus, micropatterns not fully covered by cells, replicates in which staining did not work well.
Replication	All data was obtained from at least three independent biological replicates. Some biological replicates were excluded because of poor attachment or poor staining
Randomization	Images were collected in an automated fashion at random. Samples had specific treatments and geometry confinement
Blinding	N/A it was not possible to perform blind analyses as the shapes of micropatterns revealed which samples were being analysed.

Reporting for specific materials, systems and methods

We require information from authors about some types of materials, experimental systems and methods used in many studies. Here, indicate whether each material, system or method listed is relevant to your study. If you are not sure if a list item applies to your research, read the appropriate section before selecting a response.

Materials & experimental systems

n/a	Involved in the study	
☐	☒	Antibodies
☐	☒	Eukaryotic cell lines
☒	☐	Palaeontology and archaeology
☒	☐	Animals and other organisms
☒	☐	Human research participants
☒	☐	Clinical data
☒	☐	Dual use research of concern

Methods

n/a	Involved in the study	
☒	☐	ChIP-seq
☒	☐	Flow cytometry
☒	☐	MRI-based neuroimaging

Antibodies

Antibodies used	Antibodies used for immunofluorescence are E-cadherin at :1,000 dilution (mouse mAb HECD-1, gift from Prof Takeichi) or ECCD2 at 1:750 dilution[rat mAB #13-1900, RRID:AB_2533005), anti-desmoplakin I antibody (guinea pig polyclonal #DP1, Progen, Germany)

	and myosin light chain phosphorylated at Ser19 at 1:1000 (mouse mAb #3675S, RRID:AB_2250969). Secondary conjugated antibodies were purchased from Jackson ImmunoResearch: Alexa Fluor 488 (AF488 -conjugated anti-mouse IgG (host goat, #115-545-003); Indocarbocyanine (Cy3)-conjugated anti-mouse IgG (host donkey, # 715-165-151); Indodicarbocyanine(Cy5)-conjugated anti-rat IgG (host donkey, #712-175-153); Indodicarbocyanine(Cy5)-conjugated anti-guinea pig (host goat, ##106-165-003).
Validation	All antibodies used in the experiments are commercially available and published previously. In addition, we routinely validate commercial antibodies in our laboratory using Western blots and keratinocyte lysates to check specificity, best fixative, titrations and general conditions for immuno-fluorescence for each purchased batch for staining.

Eukaryotic cell lines

Policy information about cell lines

Cell line source(s)	3T3 immortalized fibroblast were used as feeder layers to encourage keratinocyte attachment and growth. As feeder layers they are not proliferative and die before keratinocytes are used in the experiments.
Authentication	Private stock.
Mycoplasma contamination	Cells were routinely tested for mycoplasma and our stocks are mycoplasma free.
Commonly misidentified lines<br>(See ICLAC register)	N/A
